# DES-Based Biocatalysis as a Green Alternative for the l-menthyl Ester Production Based on l-menthol Acylation

**DOI:** 10.3390/molecules27165273

**Published:** 2022-08-18

**Authors:** Sabina Ion, Florentina Olănescu, Florina Teodorescu, Robert Tincu, Daniela Gheorghe, Vasile I. Pârvulescu, Mădălina Tudorache

**Affiliations:** 1 Faculty of Chemistry, University of Bucharest, 030018 Bucharest, Romania; 2 “C. D. Nenițescu” Institute of Organic and Supramolecular Chemistry, Romanian Academy, 030018 Bucharest, Romania; 3 Faculty of Applied Chemistry and Materials Science, Politehnica University of Bucharest, 030018 Bucharest, Romania; 4 Laboratory of Chemical Thermodynamics, “Ilie Murgulescu” Institute of Physical Chemistry of the Romanian Academy, 030018 Bucharest, Romania

**Keywords:** biocatalysis, DES, l-menthol, fatty acid, l-menthyl ester (FME), immobilized lipase

## Abstract

The deep eutectic solvent (DES)-based biocatalysis of l-menthol acylation was designed for the production of fatty acid l-menthyl ester (FME) using fatty acid methyl ester (FAME). The biocatalytic reaction was assisted by a lipase enzyme in the DES reaction medium. ւՒ-menthol and fatty acids (e.g., CA—caprylic acid; OA—oleic acid; LiA—linoleic acid; and LnA—linolenic acid) were combined in the binary mixture of DES. In this way, the DES provided a nonpolar environment for requested homogeneity of a biocatalytic system with reduced impact on the environment. The screening of lipase enzyme demonstrated better performance of immobilized lipase compared with powdered lipase. The performance of the biocatalytic system was evaluated for different DES compositions (type and concentration of the acid component). l-menthol:CA = 73:27 molar ratio allowed it to reach a maximum conversion of 95% methyl lauric ester (MLE) using a NV (*Candida antarctica* lipase B immobilized on acrylic resin) lipase biocatalyst. The recyclability of biocatalysts under optimum conditions of the system was also evaluated (more than 80% recovered biocatalytic activity was achieved for the tested biocatalysts after five reaction cycles). DES mixtures were characterized based on differential scanning calorimetry (DSC) and refractive index analysis.

## 1. Introduction

Menthol (*p*-menthan-3-ol) is a poorly water-soluble terpene alcohol produced from peppermint oils of *Mentha piperita* and *Mentha arvensis*. It can be found as eight optically active isomers characterized by different organoleptic properties [1,2]. l-menthol is the only isomer with refreshing coolness and specific peppermint flavor [3] widely used as a component of various kinds of foods; an ingredient of cosmetics; and an analgesic, antiseptic, and local anesthetic for pharmaceutics [4,5]. Furthermore, esters of l-menthol with fatty acids (fatty acid l-menthyl esters, FME) are known to moderate the strong flavor and to release the fragrance gradually beside the basic properties preserved from the precursors (fatty acids and l-menthol) [6]. Additionally, anti-cancer activity [7], decreasing body fat content [8], and suppressing the development of hypertension [9] are important physiological activities offered by FME. However, the developed industrial processes and the chemicals used for FME production have a negative impact on the environment [5]. In this context, green strategies for FME synthesis are requested. Enzymatic transformation (biocatalysis) is an answer to this issue due to its sustainability and low environmental impact.

Biocatalysis as an enzymatic acylation (esterification/transesterification) process for l-menthol derivatization is an efficient alternative, especially considering the green features of the transformation process [10]. Usually, biocatalysis is associated with the aqueous reaction phase required by the activity/stability of the enzyme. However, the low polarity of l-menthol and its derivatives limits their involvement in biocatalytic systems. Therefore, the reaction medium is an essential task of the biocatalytic process, particularly for l-menthol derivatization. To overcome this drawback, biocatalytic transformation of l-menthol has been performed in non-conventional reaction media such as water–oil emulsion [11], organic solvents [12], ionic liquids [5], and supercritical fluids [13]. Therefore, H. Stamatis and coworkers studied the esterification of lauric acid by l-menthol, catalyzed by *Penicillium simplicissimum* lipase in water/bis-(2-ethylhexyl)sulfosuccinate sodium salt (AOT)/isooctane microemulsions [11]. It was noticed that the microemulsions assisted in reversing the direction of lipase activity favoring synthetic reactions due to their low water content. A biocatalytic system based on the l-menthol esterification with C14/C16 fatty acids catalyzed by lipase from *Candida rugosa* has been successfully developed in dry isooctane [12]. Additionally, the effect of the low percentage of ionic liquids (e.g., 5% [BMIM][TFSI] and 1% [BMIM][BF4]) in organic MeTHF was investigated for the lipase-catalyzed kinetic resolution of *rac*-menthol [5]. Increased enantioselectivity was noticed for both cases. However, the lipase activity decreased.

In the last decade, a new class of non-conventional solvents called deep eutectic solvents (DESs) has been tested as reaction media in biocatalysis [14,15]. DES is a system prepared by combining Lewis–Bronsted acids and bases with anionic and cationic compounds, respectively. The complexation of quaternary ammonium salts with hydrogen bond donor species (e.g., amides, amines, alcohols, etc.) allows the generation of DES systems. The effect of the interaction between DES components is mostly a decrease in the freezing point of the mixture compared with the components [16]. The DES mixture is usually liquid around room temperature, and can be used as solvents for the extractions or reaction media. The DES has gained attention as solvent media, especially for biocatalysis, due to several advantages compared with other non-conventional solvents such as ionic liquids (e.g., less toxicity, biocompatibility, and biodegradability) [17]. Therefore, DES-based biocatalysis has been recently reported in the literature [18,19].

The DES mixture of menthol and carboxylic acids has been described several times [4,20]. d/l-menthol was combined with pyruvic acid/acetic acid/(-)-lactic acid/lauric acid and used further for the extraction of caffeine, tryptophan, isophthalic acid, and vanillin [17], while the combination of d/l-menthol with caprylic, capric, and lauric acids allowed the separation of neonicotinoids from diluted aqueous solutions [21]. In both cases, described DESs are stable systems in contact with water, characterized by lower vapor pressure and viscosity compared with the corresponding components, and efficient hydrophobicity compatible with nonpolar systems [22]. In the last decade, menthol-based DES was used as a source of substrate and solvent for the biocatalytic production of l-menthol derivatives [18,19]. M. Hummer et al. reported the esterification reactions of l-menthol with fatty acids (e.g., octanoic acid, decanoic acid, and lauric acid) catalyzed by lipase enzyme from *Candida rugosa* [18]. l-menthol and fatty acid were mixed previously under special conditions to provide the DES medium of the biocatalytic reaction. However, l-menthol conversion cannot reach more than 71% even after a long reaction time of 7 days. The optimization of the biocatalytic process focused on the thermodynamic water activity (aw) identified as a key parameter affecting the esterification, which allowed the process performance to be improved. Therefore, a maximum conversion of 95% was achieved for aw = 0.55 after a 24 h reaction time [19]. In the same period, A. Paiva’s group has developed DES-based biocatalysis for *rac*-menthol and lauric acid [4]. Lipase from *Candida rugosa* assisted the biocatalytic process, allowing it to reach 44% conversion of the fatty acid and 62% enantiomeric excess of the product.

In this study, we proposed a new design of the biocatalytic system for FME production based on the acylation of l-menthol with fatty acid methyl esters (FAMEs) assisted by lipase biocatalyst in a DES reaction medium. Binary mixtures of l-menthol with fatty acids were used as a DES alternative providing “green” features of the proposed system. It has been mentioned that l-menthol played the double role of a substrate for the biocatalytic acylation and also a DES component. The screening of lipase enzymes allowed us to notice the performance of the immobilized lipase compared with powdered lipase. l-menthol and different fatty acids (e.g., CA—caprylic acid, OA—oleic acid, LiA—linoleic acid, and LnA—linolenic acid) were tested to evaluate the effects of DES composition (the type and concentration of the acid component) on the biocatalytic system. Furthermore, DES characterization was performed using DSC and refractive index analysis. The effect of the FAME structure on the efficiency of the biocatalytic system was investigated in order to optimize the transesterification reaction. Biocatalyst recyclability was evaluated under optimum conditions for successive reaction cycles without washing steps.

From our knowledge, it is the first time that the biocatalytic acylation of l-menthol with FAME in DES of l-menthol and fatty acid composition has been reported. Additionally, the variety of biocatalyst designs was enriched with the immobilized lipase which exhibited good catalytic properties in the developed system, offering new biocatalyst alternatives for FME production. Currently, only *Candida rugosa* lipase is reported in the literature as the biocatalyst for DES-based FME production [18,19].

## 2. Results and Discussion

### 2.1. Lipase Screening for l-menthol Acylation

**Figure 1:**
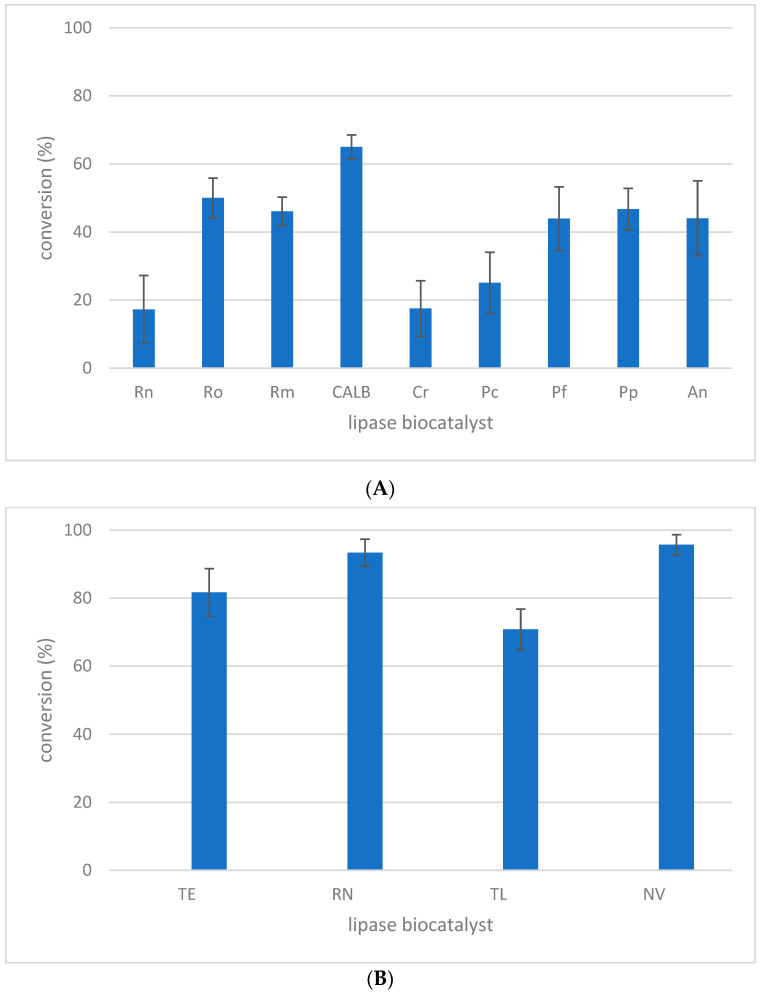
Lipase screening for l-menthol acylation with MLE in M:CA (I) DES medium using (**A**) powdered and (**B**) immobilized biocatalysists. Experimental conditions: 1.58 mM MLE, l-menthol:MLE = 3:1 molar ratio, 1 mg/mL lipase biocatalyst in M:CA (I) reaction medium, 40 °C temperature, 1000 rpm shaking, and 24 h reaction time. The values of the error bars were calculated using triplicate measurements.

Nine powdered lipases, Rn, Ro, Rm, CALB, Cr, Pc, Pf, Pp, and An, and four immobilized lipases, TE, RN, TL, and NV, were considered for the acylation of l-menthol with MLE (transesterification reaction) in DES with M:CA (I) composition (Table 1). l-menthol played the double role of the substrate and the DES component. The experimental results are presented in Figure 1A,B. For powdered lipases (Figure 1A), the process conversion was situated in the range 17–65%. The CALB lipase allowed 65% transformation of MLE to be reached, while minimum conversion (17%) was achieved by Rn and Cr. Additionally, immobilized lipase (Figure 1B) performed the MLE transesterification with higher conversion compared with powdered lipase (i.e., 70–95% conversion range determined for the immobilized lipase). Therefore, MLE conversions of 65% for CALB and 96% for NV were determined for lipase B from *Candida antarctica*. Furthermore, MLE conversions of 46% for Rm and 93% for RN were evaluated for lipase from *Rhizomucor miehei*. Similar catalytic behavior of lipase after immobilization was previously reported in the literature [23,24,25]. The preservation of this behavior for the DES reaction medium is noticeable in this case. Thus, immobilized lipase offered a good perspective as a biocatalyst for DES-based enzymatic acylation of l-menthol. Consequently, all four immobilized lipases were selected for further experiments to investigate the DES-based biocatalytic system.

### 2.2. Characterization of DES(s) Composition

Different binary mixtures of l-menthol and fatty acids (CA—C8:0, OA—C18:1, LiA—C18:2, and LnA—C18:3) were prepared based on the DES approach reported in the literature [18,20]. For l-menthol:CA/OA/LnA, the acid component was varied in the prepared mixtures. The composition of prepared mixtures is indicated in Table 1.

All of these mixtures were characterized based on DSC analysis (Figure S1 in Supporting Information). Transition temperature(s) (*T*_max_/°C) and enthalpy (Δ*H*/J g^−1^) were calculated correspondingly (Table S1 in Supporting Information). The DSC curve indicated that the melting temperature for l-menthol was around 44 °C, which is in accordance with reported data in the literature [26].

As a general remark, the temperature of the melting point(s) and also the enthalpy of the prepared mixtures were shifted to lower values compared with those of the pure components, indicating the formation of the eutectic mixture [27]. These are strong evidence of DES achievement by hydrogen bonds established between l-menthol as the acceptor component and fatty acid as the donor component [28]. M:CA (III) is the single DES solvent exhibiting only one peak of thermal effect, which demonstrates the high level of homogeneity. The enthalpy of the DES(s) decreased in the order M:CA > M:OA > M:LiA > M:LnA (Table S1 in Supporting Information). This behavior is based on the intensity of the l-menthol interaction with fatty acids affected by the structure of fatty acids. Short carbon chains of fatty acid favored the interaction with l-menthol (82.4 and 33 J·g^−1^ enthalpy of M:CA (I) and M:OA (I), respectively). Additionally, unsaturated chains of the fatty acid allowed stronger interactions with l-menthol than saturated ones (33 and 63 J·g^−1^ enthalpy of M:OA (I) and M:LiA, respectively). For different l-menthol abundances in the DES composition (M:CA (I-III) and M:OA (I-III)), mixture properties were changed randomly due to the pure homogeneity of DES when l-menthol content increased. The conclusions are also supported by the literature [20].

Additionally, the refractive index of pure compounds and their mixtures was determined at 25 °C. The data are presented in Table S2 (Supporting Information). It is known that the refractive index depends on the type of hydrogen bond donor in DES [29]. The refractive index of the DESs increased in the order M:CA < M:OA < M:LiA < M:LnA (Table S2 in Supporting Information). In the case of a binary mixture (DESs) of M:CA(I-III), the refractive index is higher than in the case of the pure components.

### 2.3. Influence of DES Composition on the Biocatalytic System

**Figure 2:**
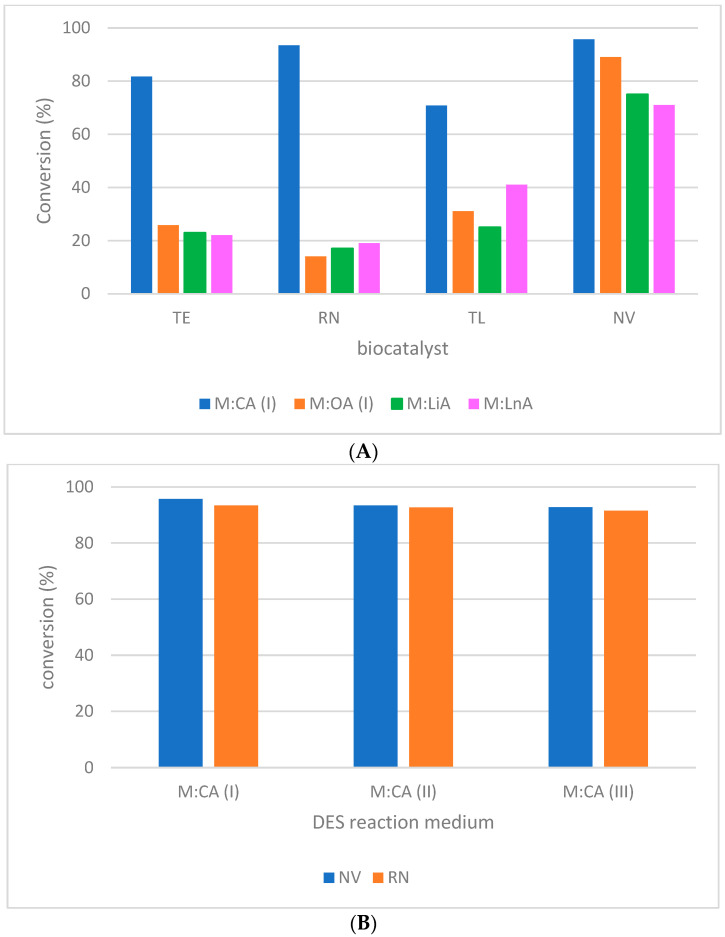
Evaluation of the performance of the biocatalytic system for different DES compositions ((**A**)—different H donor of DES and (**B**)—different concentration of the H donor in DES). Experimental conditions: 1.58 mM MLE, l-menthol:MLE = 3:1 molar ratio, 1 mg/mL lipase biocatalyst in DES reaction medium, 40 °C temperature, 1000 rpm shaking, and 24 h reaction time. Triplicate measurements of the samples with maximum 25% RSD.

Different DES compositions, based on the type and concentration of fatty acids, were tested for the acylation of l-menthol with MLE. The obtained results are inserted in Figure 2A,B. M:CA (I) offered the best reaction phase for the biocatalytic transformation (Figure 2A) with conversion >70% for all of the tested biocatalysts (TE, RN, TL, and NV). A maximum value of 95% conversion was achieved for the NV biocatalyst. The low performance of the biocatalytic system was noticed for the other DES compositions (maximum values of 89, 75, and 71% conversion for NV in M:OA (I), M:LiA, and M:LnA, respectively).

The experimental results demonstrated that the catalytic interaction of l-menthol with MLE in the acylation process was influenced by the acid (donor) type of DES composition. Therefore, CA favored the biocatalytic acylation of l-menthol, while LnA exhibited one of the intense negative effects on it. This can be explained mostly based on the -menthol solubility and also the preservation of biocatalyst activity in DES. When l-menthol was proper adapted to the reaction medium (homogeneous DES), high performance in the biocatalytic system was obtained. The conclusion is also supported by the results of DSC analysis (Section 2.2. *Characterization of DES(s) composition*). Regarding biocatalyst behavior, the NV biocatalyst exhibited better activity in the biocatalytic system compared with TE, RN, and TL for all the tested DES(s). In the literature, NV was also reported with high catalytic activity for the acylation reaction based on the transesterification mechanism in the conventional solvent medium [30]. It seems that NV performance is preserved in DES with l-menthol:fatty acid composition.

Variations in the l-menthol:CA ratio were also considered and the performance of NV and RN biocatalysts was determined in the proposed system (Figure 2B). Slight differences were observed for different DES compositions. The system performance decreased together with the component ratio. In other words, high l-menthol content favored the performance of the acylation process. DES M:CA (I) was selected for further experiments.

Negative control samples were also considered. The experimental results are inserted in Table S3 from Supporting Information. Low conversion of fatty acids was achieved, demonstrating that the immobilized lipase exhibited negligible catalytic activity for the esterification of CA/OA/LiA/LnA with l-menthol.

### 2.4. Testing the Effect of FAME on FME Production

**Figure 3:**
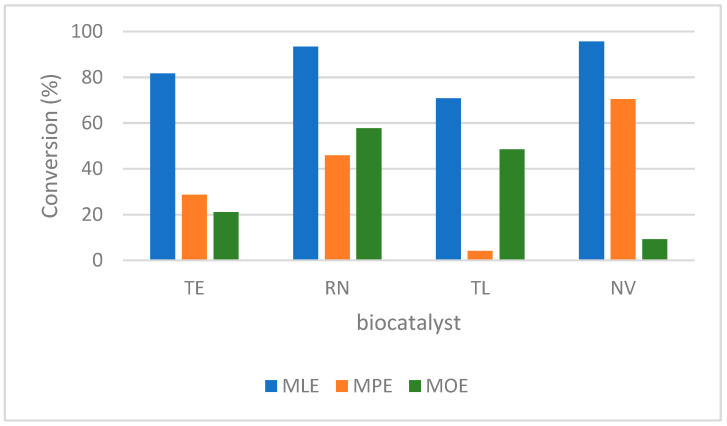
Influence of the FAME structure on the developed biocatalytic system. Experimental conditions: 1.58 mM FAME, l-menthol:FAME = 3:1 molar ratio, 1 mg/mL lipase biocatalyst in M:CA (I) reaction medium, 40 °C temperature, 1000 rpm shaking, and 24 h reaction time. Triplicate measurements of the samples with maximum 18% RSD.

Three different FAMEs (MLE, MPE, and MOE) were considered for the developed biocatalytic system. The acylation process was tested for all the immobilized lipases in DES with composition M:CA (I). The corresponding experimental data are shown in Figure 3. MLE was better recognized by the lipase biocatalyst compared with MPE and MOE. All the immobilized lipases exhibited high affinity for the short carbon chain. As an example, RN converted 93% of MLE and only 45% and 57% of MPE and MOE, respectively. This behavior was noticed for all the tested biocatalysts. The highest performance of the system was achieved for the NV biocatalyst with the MLE acylation reagent (95% conversion). Based on this, the optimum system parameters were set up as the MLE acylation reagent, the NV biocatalyst, and the M:CA (I) reaction medium, and 40 °C temperature, 1000 rpm shaking, and a 24 h reaction time were established as experimental conditions.

### 2.5. Stability of the Biocatalyst under Optimal System Conditions

**Figure 4:**
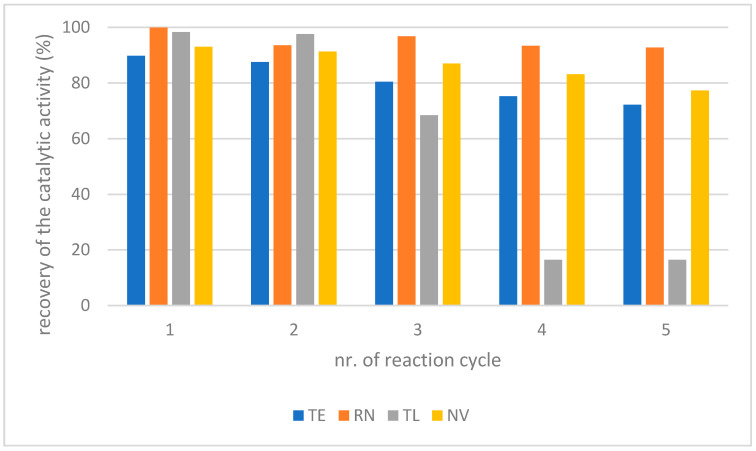
Working stability of the biocatalyst under optimum experimental conditions (1.58 mM MLE, l-menthol:MLE = 3:1 molar ratio, 1 mg/mL lipase biocatalyst in M:CA (I) reaction medium, 40 °C temperature, 1000 rpm shaking and 24 h reaction time). Experimental conditions of the determination of biocatalyst activity: 2.5 mM *p*-NPB dissolved in ethanol, 1:4 *v*/*v* protein extract, and 32.5 mM Tris-HCl (pH 7.2) was incubated for 30 min at 37 °C, and the reaction was terminated by addition of 20 mM Na_2_CO_3_ blocking solution (10 min incubation). Triplicate measurements of the samples with maximum 23% RSD.

The working stability of the biocatalyst was tested for all of the immobilized enzymes (TE, RN, TL, and NV). The biocatalysts were used for five successive reaction cycles without any intermediary washing steps. The recovered biocatalyst activity was calculated and related to the initial catalytic activity (Figure 4). RN and NV exhibited the best stability under the experimental conditions. The catalytic activity of RN/NV lost less than 20% after five reaction cycles. Oppositely, TL exhibited low stability (only 16% recovered catalytic activity was noticed after five reaction cycles).

## 3. Experimental

### 3.1. Chemicals and Solutions

Lipase from *Rhizopus niveus* (Rn), *Rhizopus oryzae* (Ro), *Rhizomucor miehei* (Rm), *Candida rugosa* (Cr), *Aspergillus niger* (An), *Pseudomonas fluorescens* (Pf), *Pseudomonas cepacia* (Pc), Porcine pancreas (Pp), and lipase B from *Candida antarctica* (CALB) (Sigma-Aldrich, Sofia, Bulgaria) were used as powder dispersed in the DES medium to catalyze the acylation (transesterification) process. Novozym 435 (Ca lipase B immobilized on acrylic resin, NV), Lipozyme TL (*Thermomyces lanuginosus* lipase immobilized on silica gel carrier) (TL), Lipozyme RM (Rm lipase immobilized on resin carrier) (RN) from commercial sources (Novozymes A/S, Bagsvaerd, Denmark), and Transenzyme (lipase from *Geobacillus stearothermophilus* immobilized in sol–gel matrices) [31] (TE) prepared in the lab of Prof. A. Fishman (Technion–Israel Institute of Technology, Haifa, Israel) were the immobilized biocatalysts tested in the FME system. l-menthol was provided by our partner Natural Ingredients SA (Fagaras, Romania) in the frame of the PED376/2020 research project. Fatty acids (CA—C8:0, OA—C18:1, LiA—C18:2, and LnA—C18:3) and FAMEs (methyl laurate ester—MLE, methyl palmitate ester—MPE, and methyl oleate ester—MOE) were purchased from Sigma-Aldrich (Sofia, Bulgaria) and the Merck (Bucharest, Romania) company, respectively. For HPLC analysis, acetonitrile (ACN) and acetone (Act) of analytical purity grade were purchased from the Sigma-Aldrich company (Sofia, Bulgaria).

### 3.2. DES Preparation

The DESs, as a binary mixture of l-menthol and fatty acids, such as l-menthol:caprylic acid (M:CA I, II and III), l-menthol:oleic acid (M:OA I and II), l-menthol:linoleic acid (M:LiA), and l-menthol:linolenic acid (M:LnA), were prepared by adding the components into glass vessels at a certain molar ratio, as indicated in Table 1. The mixtures were incubated at 40 °C under stirring at 250 rpm overnight until the homogeneous phase was obtained, and then cooled to room temperature. [20].

### 3.3. DES Characterization

The thermal behavior of DESs was investigated *via* differential scanning calorimetry (DSC) using a power-compensated calorimeter from PerkinElmer, USA (model 8500) with a cooling system (Intracooler III). Sealed aluminum pans were used for the DES samples and standards, ideally for volatile samples, while an empty pan was used as a reference. Calibration of the temperature and the heat flow rate scale was performed by measuring high-purity indium (*T_fus_* = 156.7 °C and Δ*H_fus_* = 28.5 J g^−1^). The DSC curves of the studied compounds were recorded under nitrogen (> 99.996% purity) with a flow rate of 20 mL min^−1^. Samples were scanned from 25 to −30 °C at 10 °C min^−1^, held at −30 °C for 2 min before heating to 60 °C at 10 °C min^−1^. The thermal effects (the temperature of crystallization, the melting point, and the enthalpy of the system) were calculated using Pyris Software (V 11, PerkinElmer, Waltham, MA, USA) for Windows.

The refractive indices of prepared DESs were carried out at sodium D-line at λ_D_ = 589.3 nm, using a digital automatic refractometer (Anton Paar RXA 170) with accuracy of ± 0.01 K for temperature and ± 0.000001 for the refractive index. The refractometer was calibrated using double distilled water. An average of triplicate measurements was considered for each sample [32]. Certified reference liquid (CRM) tetrachloroethylene was used for the calibration. Further, the system checked out with deionized water at atmospheric pressure. The refractive index measured for water (n_D_^20^ = 1.33302 ± 0.00003) was similar with the value reported in the literature (n_D_^25^ = 1.33249) [33].

### 3.4. Biocatalytic Approach for l-menthol Acylation

A mass of 1 g DES was weighed into a 1.5 mL reaction tube where 1 mg of lipase and amounts of FAME were added to prepare a reaction mixture with l-menthol:FAME = 3:1 molar *ratio*. A negative control was prepared for each DES-containing enzyme in the absence of FAME. The reaction mixture was incubated for 24 h at 40 °C under stirring conditions (1000 rpm). After the reaction, the sample was centrifugated and the supernatant was diluted 1:10 with a solution of ACN:Act = 41:59, *v*/*v* (mobile phase of HPLC analysis), after filtration (0.2 μm pore size).

HPLC analysis based on DAD and RID detection (HPLC-DAD/RID) was performed for the determination of sample composition after the biocatalytic acylation of l-menthol. The modular HPLC system (Agilent 1260) equipped with C18 column (Europhore 100-5, L × d = 250 × 4 mm, 5 μm particles size) was used for the analysis. The HPLC-DAD/RID was set up for a 25 μL sample volume, a 1 mL/min flow rate of the mobile phase (ACN:Act = 41:59, *v*/*v*), and a temperature of 25 °C in the column thermostat. Detection was performed with DAD at 210 nm and RID at 40 °C. The identification and quantitative determination of the sample components (l-menthol, fatty acid, FAME and FME) were achieved based on the calibration curve of corresponding standards. Based on the HPLC analysis results, FAME conversion was calculated according to the equation inserted bellow. FMEs (menthyl laurate, menthyl palmitate, and menthyl oleate) synthesized and characterized in our lab (SI) were used as reference materials for the identification of the l-menthol esters in the chromatograms.

(1) $$C\;\left(\%\right)=\frac{\mathrm{FAME}\;\mathrm{mass}\;\mathrm{after}\;\mathrm{reaction}}{\mathrm{initial}\;\mathrm{mass}\;\mathrm{of}\;\mathrm{FAME}}\times 100$$

### 3.5. Evaluation of the Biocatalyst Recyclability

Immobilized lipases (TE, RN, TL and NV) were tested for five successive reaction cycles in the developed biocatalytic system. The following experimental conditions were set up: 1.58 mM of MLE, l-menthol:MLE = 3:1 molar ratio, 1 mg/mL of TE/RN/TL/NV in the M:CA (I) reaction medium, 40 °C temperature, 1000 rpm shaking, and a 24 h reaction time. After each reaction cycle, the biocatalyst was recovered from the reaction phase and its catalytic activity was determined using the *p*-NPB method [34]. The recovered biocatalyst activity was calculated as the percentual value of the ratio between catalytic activity after the reaction cycle and the initial catalytic activity.

## 4. Conclusions

DES-based biocatalysis for FME production was developed using l-menthol acylation with FAME assisted by a lipase enzyme. The DES reaction medium was used with l-menthol as the donor and fatty acids as the acceptor components, providing proper homogeneity to the biocatalytic system. Immobilized lipase exhibited better catalytic performance compared with powdered lipase in the proposed system. In this way, a new biocatalyst for FME production using DES-based biocatalysis was discovered, since only lipase from *Candida rugosa* has been reported in the current literature [18,19]. Under the optimal experimental conditions, 95% MLE was converted using NV biocatalyst and M:CA (I) DES at 40 °C temperature, 1000 rpm shaking, and a 24 h reaction time. Additionally, the biocatalyst can be used over at least five consecutive reaction cycles by preserving the catalytic activity (recovered catalytic activity >80%).

The developed system has several advantages compared with the literature-reported alternatives for FME production: (i) it allows us to perform an efficient biocatalytic transformation of FAME into FME (e.g., 95% conversion for MLE); (ii) it provides a nonpolar reaction medium proper for the solubilization of reagents/products in the reaction phase, preserving the biocatalyst activity; (iii) it exhibits versatility by easily adapting to different FAME (e.g., MLE, MPE, and MOE) for optimum biocatalyst—DES couple; (iv) it is cost-effective based on the use of immobilized lipase allowing the recyclability and reusability of the biocatalyst (more than 80% recovered catalytic activity of RN/NV for five reaction cycles); and (v) it has a low impact on the environment due to green characteristics of the system components and experimental conditions. Besides all of these, the developed biocatalytic system is a good perspective for future FME production in lab with impact at an industrial scale.

## Figures and Tables

**Table 1:** DES composition based on l-menthol and fatty acid components.

	Molar Ratio (%) of Acceptor vs. Donor
M:CA (I) ^1^	73:27
M:CA (II) ^1^	65:35
M:CA (III) ^1^	50:50
M:OA (I) ^2^	83:17
M:OA (II) ^2^	62:38
M:OA (III) ^2^	50:50
M:LiA ^3^	83:17
M:LnA ^4^	83:17

DES donor: ^1^ CA, ^2^ OA, ^3^ LiA, and ^4^ LnA. l-menthol was the DES acceptor in all the cases.

## Supplementary Materials

The following supporting information can be downloaded at: https://www.mdpi.com/article/10.3390/molecules27165273/s1. Figure S1: Comparative DSC scans obtained at heating rate 10 °C min^−1^ of mixtures of l-menthol (M) and fatty acids (CA—C8:0, OA—C18:1, LiA—C18:2, and LnA—C18:3). Table S1: Characterization of DES(s) using DSC analysis. Table S2: Comparative data of refractive indices (nD) at 25 °C of pure compounds and DES mixtures [1,2,3,4,5,6]. Table S3: Testing the catalytic activity of the immobilized lipases (TE/RN/TL/NV) for the reaction of DES components—the determination of fatty acid conversion (%) based on the acylation of l-menthol with DES donor (CA, OA, LiA and LnA). Experimental procedure: FME synthesis; FME characterization.
