# Supplementary material for: DES-Based Biocatalysis as a Green Alternative for the l-menthyl Ester Production Based on l-menthol Acylation
Source: Molecules. 2022 Aug 18;27(16):5273. doi: 10.3390/molecules27165273 (PMC9412368; doi:10.3390/molecules27165273)
Supplement: Supplementary file 1 [file molecules-27-05273-s001.zip › molecules-1837315-supplementary.pdf]

Supplementary

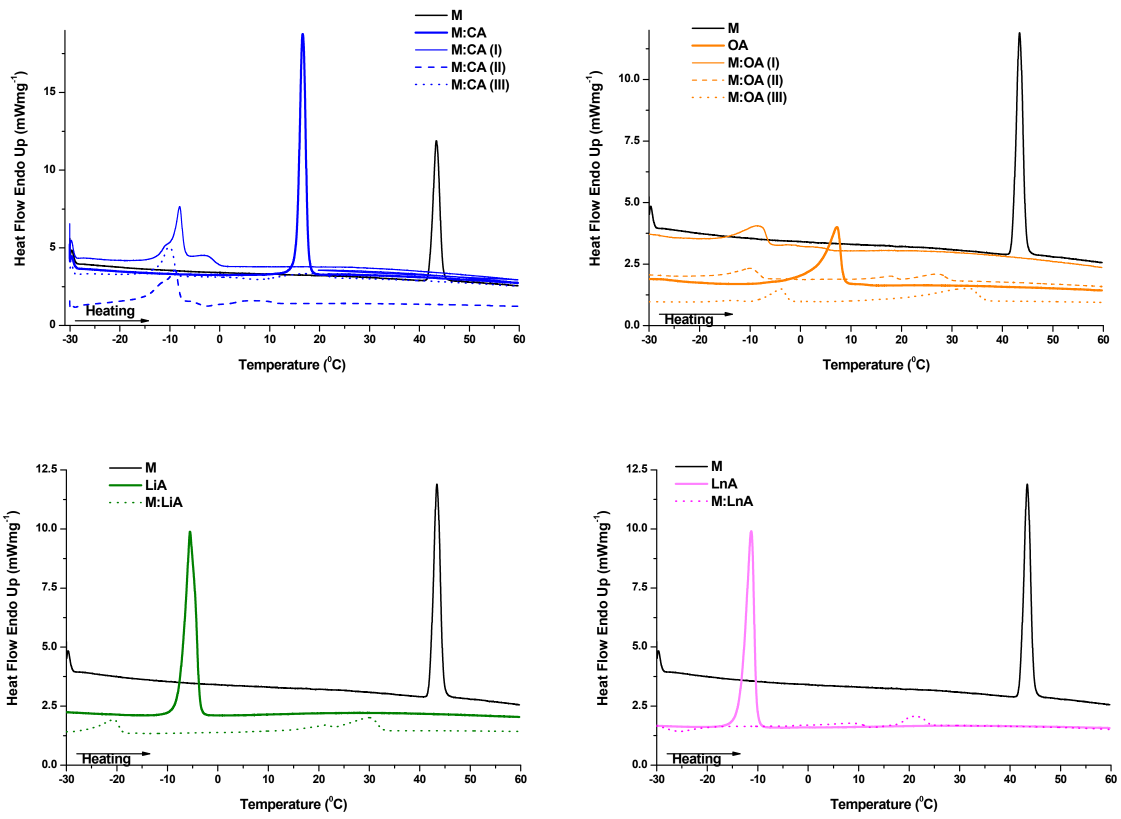

Figure S1. Comparative DSC scans obtained at heating rate 10 ºC min^{-1} of mixtures of ʟ-menthol (M) and fatty acids (CA - C8:0, OA - C18:1, LiA - C18:2, and LnA - C18:3)

Table S1. Characterization of DES(s) using DSC analysis.

Sample	Ramp	Process	T_i/ºC	T_{max} /ºC	T_f/ºC	ΔH/J g^{-1}
ʟ-menthol	cooling	-	-	-	-	-
	heating	melting	42.1	43.4	44.6	87.7
CA	cooling	crystallization	6.3	6.0	5.3	-143.4
	heating	melting	15.4	16.6	17.8	144.1
M:CA (I)	cooling	crystallization	-20.9	-23	-24.9	-84.3
	heating	melting	-9.9	-8.0	6.7	82.4
M:CA (II)	cooling	-	-	-	-	-
	heating	melting	-13	-9.0	-7.7	93.9
			1.0	5.7	9.5	14.1
M:CA (III)	cooling	crystallization	-3.0	-5.2	-9.5	-10.9
	heating	melting	-12.4	-10.1	-8.7	34.7
			9.3	18.1	22.6	21.6
OA	cooling	crystallization	-8.5	-10.0	-11.7	-68.3
			-34.2	-38.0	-42.0	-6.15
	heating	melting	-34.0	-27.7	-24.2	-1.2
			3.2	7.2	8.5	67.4
M:OA (I)	cooling	crystallization	-28.3	-30.9	-33.4	-12.5
	heating	melting	-45.9	-44.3	-42.6	-12.8
			-12.7	-8.4	-6.3	33.0
M:OA (II)	cooling	crystallization	7.7	2.8	0.4	-12.3
			-28.5	-31.7	-34.0	-4.7
			-36.6	-41.5	-44.6	-6.9
	heating	melting	-18.1	-10.0	-7.7	12.6
			4.1	18.0	19.4	3.8
			24.7	27.3	29.6	8.1
M:OA (III)	cooling	crystallization	16.9	15.3	13.5	-1.0
			10.3	8.6	6.5	-1.3
			-20.2	-24.2	-29.5	-16.4
	heating	melting	-17.5	-13.4	-11.0	1.3
			-8.1	-3.9	2.2	11.4
			22.1	33.2	36.3	37.9
LiA	cooling	crystallization	-26.4	-27.2	-28.0	-111.8
			-62.2	-64.2	-66.8	-3.5
	heating	melting	-58.8	-56.5	54.7	4.8
			-7.4	-5.5	-36	117.9
M:LiA	cooling	crystallization	10.1	9.1	7.6	-143.3
			-62.1	-63.6	-65.9	-27.02
	heating	melting	-25.6	-20.7	-18.9	63.02
			0	29.9	32.6	118.9
LnA	cooling	crystallization	-19.6	-21.1	-23.2	-102.0
	heating	melting	-13.4	-11.3	-10.3	104.3
M:LnA	cooling	-	-	-	-	-
	heating	-	-31.5	-25.8	-19.6	-37.1
		melting	6.6	8.6	11.9	8.8
			17.7	21.1	24.3	12.5

Table S2. Comparative data of refractive indices (n_D) at 25ºC of pure compounds and DES mixtures.

sample	n_D^{25}±SD (this work)	n_D^{25} (literature)
L-menthol	–	1.3284 [1]
CA	1.42603±0.00016	1.4258 [2]
M:CA (I)	1.44344±0.00012	–
M:CA (II)	1.44887±0.00023	–
M:CA (III)	1.4517±0.00032	–
OA	1.46195±0.00011	1.4577 [3]
M:OA (I)	1.46114±0.00030	–
M:OA (II)	1.46043±0.00035	–
M:OA (III)	–	–
LiA	1.46803±0.00027	1.468-1.472 [4]<br>1.478 [5]
M: LiA	1.46241±0.00029	–
LnA	1.47903±0.00021	1.480 [6]
M: LnA	1.46322±0.00022	–

* SD-standard deviation of mean for triplicate consecutive measurements

Tabel S3. Testing the catalytic activity of the immobilized lipases (TE/RN/TL/NV) for the reaction of DES components - the determination of fatty acid conversion (%) based on the acylation of L-menthol with DES donor (CA, OA, LiA and LnA).DES . The control did not contain lipase. (Triplicate measurements of the samples with maximum 20 % RSD)

	TE	RN	TL	NV	control
CA	2.5	2.9	0.7	0.5	-
OA	1.4	0.1	0.8	0.2	-
LiA	0.5	0.1	0.3	1.2	-
LnA	0.3	0.1	0.5	0.8	-

Experimental procedure

FME synthesis

The menthyl esters of fatty acids were synthesized adapting Neises's method (B. Neises, W. Steglich, Simple Method for the Esterification of Carboxylic Acids, Angew. Chem., 17 (1978), 522-524). 0.01 mol of fatty acid and 0.001 mol of N, N-dimethylaminopyridine (DMAP) were dissolved in 25 mL of methylene chloride and then 0.01 mol of menthol were added. The solution was cooled to 0 °C followed by the addition of 0.01 mol of N,N-dicyclohexylcarbodiimide (DCC). The resulted mixture was stirred at 0 °C for 5 minutes and then at room temperature for 3h. Consequently, the formed precipitate was removed by the filtration and the supernatant was dried under reduced pressure (10 mmHg) leading to a residue. The residue was dissolved in a small amount of methylene chloride, and any additionally formed precipitate was removed through filtration. The solution was washed twice with 25 mL of hydrochloric acid 0.5 N and dried under reduced pressure. The crude product was purified by column chromatography on silica gel, elution was performed with EE/EP gradient mixtures, and collecting the fraction eluted with 1:9 ratio of solvents. Evaporation of the solvent gave an analytically pure compound (FME).

FME characterization

The solid materials were characterized by FTIR spectroscopy with a Bruker VERTEX 70 instrument, equipped with a Harrick MVP2 diamond ATR device. Also, NMR spectra were recorded on a Bruker Advance III 600 MHz spectrometer, corresponding to the resonance frequency of 600.12 MHz for the ^{1}H nucleus, equipped with an indirect detection for nuclei probe head (BBI) and field gradients on Z axis. Samples were analyzed in 5 mm NMR tubes (Willmad).

Menthyl palmitate (2.03g, Rf=0.65) ^{1}H-NMR (600 MHz, Chloroform-*d*) δ 4.66 (td, J = 10.9, 4.4 Hz, 1H), 2.25 (td, J = 7.3, 1.4 Hz, 2H), 1.96 (dd, J = 12.0, 2.1 Hz, 1H), 1.85 (td, J = 7.0, 2.8 Hz, 1H), 1.70 – 1.62 (m, 2H), 1.62 – 1.53 (m, 2H), 1.52 – 1.42 (m, 1H), 1.39 – 1.32 (m, 1H), 1.31 – 1.20 (m, 24H), 1.11 – 1.00 (m, 1H), 0.97 – 0.89 (m, 1H), 0.90 – 0.78 (m, 10H), 0.74 (d, J = 7.0 Hz, 3H). ^{13}C-NMR (151 MHz, Chloroform-*d*) δ 173.39, 73.81, 47.02, 40.95, 34.73, 34.28, 31.66, 31.35, 29.68, 29.57, 29.47, 29.41, 29.38, 29.35, 29.20, 29.07, 28.90, 26.22, 25.12, 24.70, 23.40, 22.56, 21.99, 20.72, 16.25, 14.02. FTIR: 2925, 2862, 1731, 1458, 1374, 1249, 1168, 1105, 981.

Menthyl laurate (2.53g Rf=0.72) ^{1}H-NMR (600 MHz, Chloroform-*d*) δ 4.67 (td, J = 10.9, 4.4 Hz, 1H), 2.32 – 2.22 (m, 2H), 1.98 (dd, J = 12.1, 2.0 Hz, 1H), 1.86 (td, J = 7.0, 2.8 Hz, 1H),

1.67 (ddq, J = 13.3, 6.7, 3.2 Hz, 2H), 1.63 – 1.57 (m, 2H), 1.48 (dtd, J = 12.0, 5.9, 3.1 Hz, 1H), 1.40 – 1.33 (m, 1H), 1.27 (d, J = 22.7 Hz, 16H), 1.05 (dd, J = 12.9, 3.5 Hz, 1H), 0.95 (q, J = 11.8 Hz, 1H), 0.91 – 0.82 (m, 10H), 0.75 (d, J = 7.0 Hz, 3H). ^{13}C-NMR (151 MHz, Chloroform-*d*) δ 173.47, 73.87, 47.05, 40.98, 34.78, 34.30, 31.91, 31.38, 29.61, 29.59, 29.49, 29.33, 29.27, 29.14, 26.26, 25.15, 23.44, 22.68, 22.02, 20.76, 16.29, 14.10. FTIR: 2923, 2859, 1732, 1458, 1374, 1245, 1177, 1107, 981.

Menthyl oleate (2.55g, Rf=0.73) ^{1}H-NMR (600 MHz, Chloroform-*d*) δ 5.34 (qd, *J* = 3.8, 1.8 Hz, 2H), 4.67 (td, *J* = 10.9, 4.4 Hz, 1H), 2.27 (td, *J* = 7.3, 1.1 Hz, 2H), δ 2.03 – 1.95 (m, 4H), 1.86 (td, *J* = 7.0, 2.8 Hz, 1H), 1.67 (ddd, *J* = 13.6, 6.5, 2.9 Hz, 2H), 1.63 – 1.58 (m, 2H), 1.48 (ddt, *J* = 8.7, 5.3, 2.8 Hz, 1H), 1.34 (d, *J* = 2.9 Hz, 2H), 1.33 – 1.24 (m, 20H), 1.05 (dd, *J* = 12.8, 3.4 Hz, 1H), 0.98 – 0.91 (m, 1H), 0.91 – 0.86 (m, 10H), 0.75 (d, *J* = 7.0 Hz, 3H). ^{13}C-NMR (151 MHz, Chloroform-d) δ 173.43, 129.98, 129.75, 73.86, 47.03, 40.96, 34.75, 34.29, 31.90, 31.37, 29.76, 29.68, 29.52, 29.32, 29.31, 29.16, 29.12, 29.11, 27.21, 27.17, 26.24, 25.13, 23.42, 22.68, 22.02, 20.76, 16.28, 14.10. FTIR: 3005, 2954, 2923, 2854, 1732, 1457, 1370, 1243, 1176, 1097, 1011, 985.
